# The association between sugar-sweetened beverages and milk intake with emotional and behavioral problems in children with autism spectrum disorder

**DOI:** 10.3389/fnut.2022.927212

**Published:** 2022-08-04

**Authors:** Si Tan, Ning Pan, Xiaoyu Xu, Hailin Li, Lizi Lin, Jiajie Chen, Chengkai Jin, Shuolin Pan, Jin Jing, Xiuhong Li

**Affiliations:** ^1^Department of Maternal and Child Health, School of Public Health, Sun Yat-sen University, Guangzhou, China; ^2^Guangdong Provincial Engineering Technology Research Center of Environmental Pollution and Health Risk Assessment, Department of Occupational and Environmental Health, School of Public Health, Sun Yat-sen University, Guangzhou, China

**Keywords:** sugar sweetened beverages, milk, autism spectrum disorder, emotional and behavioral problems, children

## Abstract

**Background:**

Emotional and behavioral problems are common in children with autism spectrum disorder (ASD). It's still unclear whether children with ASD have abnormal sugar-sweetened beverages (SSBs) and milk intake and whether this abnormality will affect their emotions and behavior remains unclear. The current study aims to investigate the association of SSBs and milk intake with emotional and behavioral problems in children with autism spectrum disorder (ASD).

**Methods:**

107 children with ASD and 207 typical developing (TD) children aged 6-12 years old were recruited for the study. The frequency of SSBs and milk intake was assessed by a self-designed questionnaire. Emotional and behavioral problems were assessed by Strength and Difficulties Questionnaire (SDQ). Then, the linear regression model was produced to evaluate the association of SSBs and milk intake with emotional and behavioral problems.

**Results:**

In the current study, there was no difference in frequency of SSBs intake between children with ASD and TD children (*p* > 0.05), and children with ASD consumed less milk compared to TD children (*p* < 0.05). After adjusting sex, age, maternal and paternal education, and monthly family income, we found a significant difference in each subscale score of SDQ in the two groups (*p* < 0.05). In children with ASD, higher frequent SSBs intake was positively associated with the scores of the emotional problem (*p* for trend <0.05), and lower frequent milk intake was inversely associated with the scores of prosocial behavior (*p* for trend <0.05). No interactive effects were found on SSBs and milk intake with emotional and behavioral problems (*p* for trend > 0.05).

**Conclusion:**

In children with ASD, frequency of SSBs and milk intake was associated with the emotional problem and prosocial behavior, respectively. Children with ASD should increase the frequency of milk intake and decrease the frequency of SSBs intake.

## Introduction

Autism spectrum disorder (ASD) is a serious neurodevelopmental disorder characterized by social communication deficits and repetitive behaviors (1). Children with ASD are more likely to develop substantial comorbidities, including emotional (e.g., depression and anxiety mood) and behavioral (e.g., hyperactivity and opposed behavior) problems (2). Literature showed that 72~86% of children with ASD had at least one emotional or behavioral problem (3). Emotional and behavioral problems may discourage the development of social communication and interaction in children with ASD. Moreover, it also leads to isolation, interferes with educational and therapeutic intervention, and limits participation in social and community activities (4). Therefore, emotional and behavioral problems are non-negligible problems in children with ASD.

Interestingly, some studies showed that there was a relationship between SSBs intake with emotional and behavioral problems in typically developing (TD)children (5), such as depression (6), and hyperactivity/inattention (7). Sugar-sweetened beverages (SSBs) refer to beverages with added artificial sugar or sugar content of more than 5% added in the process, such as tea drinks, carbonated drinks, sweetened milk tea, coffees, sweetened fruit juices, and sports drinks (8). It has been well documented that SSBs intake has harmful effects on children health, such as obesity, dental caries, high blood pressure, metabolic syndromes, diabetes and cardiovascular diseases (9), and emotional and behavioral problems (5). Some studies have shown that compared to TD children, children with ASD tend to intake more SSBs and sugars due to refuse bitter taste during the period of preschool and primary school (10, 11). Unfortunately, children with ASD were vulnerable to diet (12), so we speculated that higher frequent SSBs intake was positively associated with emotional and behavioral problems in the children with ASD.

In addition, literature studies have indicated that SSBs intake was associated with milk intake (13), but the association was inconsistent. Some studies showed that increased SSBs intake was associated with reduced milk intake in TD children aged 3–13 years (14–16), while another study showed that SSBs intake was positively associated with milk intake among low family income preschool-age children (17). Milk is considered beneficial for child growth and development as it is an excellent source of essential nutrients (18). Previous studies showed that milk intake might reduce the risk of chronic diseases such as overweight and obesity (19), metabolic syndrome (20), high-blood pressure (21), as well as improve bone health (22) and sleep satisfaction (23). A few studies showed that compared to TD children, children with ASD aged 4–10 years have less frequent milk intake (24, 25). Two studies indicated that meeting recommendations for milk and alternative in childhood was associated with fewer health care encounters for mental illnesses, such as depressive episodes, mood disorder, general anxiety disorder, attention-deficit/hyperactivity disorder (ADHD), and so on (26, 27). Therefore, it's reasonable to speculate that milk intake was negatively associated with emotional and behavioral problems, and there were interactive effects between SSBs and milk intake on emotional and behavioral problems in children with ASD.

In summary, the present study aimed to explore: (1) compared TD children, whether children with ASD had a different frequency of SSBs and milk intake; (2) whether there was an association between SSBs and milk intake with emotional and behavioral problems in children with ASD; (3) whether there were interactive effects between SSBs and milk intake on emotional and behavioral problems in children with ASD.

## Materials and methods

### Study population and procedure

The data in the current study were from the baseline data of an ongoing study “the Guangzhou Longitudinal Study of Children with ASD”, which examined the developmental trajectories of 6- to 12-year-olds children with ASD in Guangzhou, China. In this study, the children with and without ASD were diagnosed by the Childhood Autism Rating Scale (CARS), and two professional child psychiatrists further confirmed their diagnosis using the Diagnostic and Statistical Manual of Mental Disorders, Fifth Revision (DSM-5) criteria. The additional inclusion criteria for both groups were as follows: (1) chronological age was from 6 years 0 months to 12 years 11 months; (2) children's parents voluntarily participated in the study; (3) all participants reported without dyslexia, seizures, head trauma, cerebral palsy, or other movement disorders; (4) all participants reported without known genetic or chromosomal abnormalities or severe visual or hearing impairment. All children came from different families. Finally, a total of 107 children with ASD (90 boys and 17 girls) and 207 TD children (113 boys and 94 girls) were enrolled in this study (the flow chart of subject screening is shown in Supplementary Figure S1).

### Sugar-sweetened beverages and milk intake

The question about SSBs and milk frequency was designed according to a parent-reported Food Frequency Questionnaire (FFQ). The average Intraclass Correlation Coefficient (ICC) for test-rest reliability of the questionnaire was 0.398 (28). The questions included: “In the past 7 days, how many times did your child drink SSBs/milk?” if they answered more than one time, they were further asked about cups of SSBs/milk each time consumed from the question “How many cups of SSBs/milk did your child drink per time on average? (One cup is equal to 250 mL)”. All of these questions were responded to by parents of children. Total weekly SSBs/milk intake was calculated as (weekly times of SSBs or milk intake) × (cups of SSBs or milk intake each time). Finally, the frequency of SSBs intake was categorized as: “0 times/week”, “1-2 times/week”, “> 2 times/week”, and the frequency of milk intake was also categorized as, “0-3 times/week”, “4-7 times/week”, “>7 times/week”.

### Strengths and difficulties questionnaire

The parent-reported Strengths and Difficulties Questionnaire (SDQ) was used to assess children's emotional and behavioral problems (29). The parent-reported SDQ is widely used to screen multidimensional mental health for children aged 3–16 years. The SDQ consists of 25 items and is divided into five sub-scales, including emotional symptoms, conduct problems, hyperactivity/inattention, peer relationship problems, and prosocial behavior. Based on the child's performance in the last 6 months, each item was answered on a three-point response scale (0= not true,1= somewhat true, 2= certainly true). The higher scores for difficult parts mean having serious emotional and behavioral problems, except for the prosocial behavior subscale, where lower scores indicated greater difficulties. Previous studies demonstrated that the internal consistency of the sub-scales of SDQ was good (Cronbach's α, 0.50–0.74) (30).

### Covariates

The covariates were collected in the current study, including individual factors and family factors (31). Individual factors included sex (boys and girls), age (years), physical activity (intensity of exercise: high, moderate, low), full scale intelligence quotient (FSIQ) (score), screen times (<2h/day, > 2h/day), body mass index (BMI) (underweight, normal, overweight); family factors included maternal education (high school or below, university or above), paternal education (high school or below, university or above) and monthly family income (<8,000 RMB/month, >8,000 RMB/month), which indicated the family socioeconomic status. Owing to the “females protect effect”, where females would require the greater etiologic load to manifest the same degree of affectedness as males (32), females had lower autism prevalence than males (33). Thus, we controlled the sex as covariates in the following analysis. All of the demographic information was collected from the children's parents. The FSIQ of the participants was measured by the Chinese version of the Wechsler IV Intelligence Test (34).

Physical activity was assessed by the International Physical Activity Questionnaire (IPAQ) short format (35). All participants were asked to report the days and times in which they performed physical activity during the last 7 days at three intensities: high, moderate, and low. Examples of high activities like heavy lifting, digging, aerobics, or fast bicycling, moderate activities included carrying light loads, bicycling at a regular pace or doubles tennis, and low activities included walking. Metabolic Equivalent of Tasks (METs) is a standard unit of measurement for expressing the intensity of physical activity. The total weekly physical activity was calculated as a continuous variable by weighting the time performing each activity intensity with its metabolic equivalent (MET). Based on the total METs, the participants were classified into low (0 ≤ METs <600), moderate (600 ≤ METs <3000), and high (METS ≥ 3,000) groups.

The BMI was estimated by dividing weight (kg) by height^2^ (m^2^), and the measurements of weight and height were taken by trained staff according to the standard procedure of anthropometric procedures and data collection developed by the World Health Organization (WHO). We adopted the age- and gender-specific BMI cutoff points for Chinese school-age children and adolescents released by the National Health and Family Planning Commission in 2018, underweight and overweight were defined as ≤ 5th percentile and ≥85th percentile, then classified BMI into underweight, normal, and overweight (36).

### Statistical analysis

R core team version 4.0. was used for the statistical analysis. Data were presented as mean ± standard deviation (SD) for continuous variables and percentage for categorical variables. Next, in order to assess the differences between the children with ASD and TD children, the two-sided *t* test and chi-square tests were used for consecutive data and categorical variables, respectively (37). Then, we adopted a linear regression model to construct three models to analyze the association of SSBs, milk intake, and their interactive effect on SDQ sub-scales. Owing to the interactive effects of SSBs intake within the group, milk intake in the group was significant, we further analyzed the association of SSBs intake and SDQ sub-scales, milk intake, and SDQ sub-scales in the group of children with ASD and TD children respectively. The 0 time/week and 0–3 times/week were, respectively, treated as a reference for SSBs and milk intake, and model estimates were presented by estimates (β) and 95% confidence interval (CI). We performed trend tests by entering the median value of SSBs/milk intake as continuous variables in the models. The adjusted model included demographic factors, such as sex, age, physical activity, FSIQ, screen times, BMI, family income, and maternal and paternal education. All criteria for statistical significance were set at a two-tailed *p* < 0.05.

## Result

### The demographic characteristics of all participants

Table 1 summarized the demographic characteristics of the participants. From a total of 314 participants, 107 were diagnosed with ASD (90 boys, 84.11%) and 207 were normal controls (113 boys, 54.59%). Compared to children with TD, children with ASD were more likely to be boys, a mother with lower education degrees, a family with lower monthly incomes, lower FSIQ, longer screen time, and lower frequent physical activity (all *p* < 0.05).

**Table 1:** Demographic characteristics of the study participants.

	**ASD (*N =* 107)**	**TD (*N =* 207)**	**Total (*N =* 314)**	***p-*value**
**Age, y**	7.72 ± 1.30	7.77 ± 1.32	7.75 ± 1.31	0.791
**Sex, %**				<0.001
Boys	90 (84.11)	113 (54.59)	203 (64.65)	
Girls	17 (15.89)	94 (45.41)	111 (35.35)	
**Maternal education, %**				0.003
High school or below	44 (41.12)	51 (24.64)	95 (30.25)	
University or above	63 (58.88)	156 (75.36)	219 (69.75)	
**Paternal education** ^a^ **, %**				0.054
High school or below	40 (37.38)	73 (35.44)	113 (36.10)	
University or above	67 (62.62)	133 (64.56)	200 (63.90)	
**Monthly family income, %**				<0.001
< RMB 8000	61 (57.01)	52 (25.12)	113 (35.99)	
≥RMB 8000	46 (42.99)	155 (74.88)	201 (64.01)	
**FSIQ** ^b^	92.01 ± 18.76	112.88 ± 12.77	106.51 ± 17.67	<0.001
**BMI** ^c^ **, %**				0.059
Underweight	10 (9.35)	26 (12.75)	36 (11.58)	
Normal	68 (63.55)	146 (71.57)	214 (68.81)	
Overweight	13 (12.15)	19 (9.31)	32 (10.29)	
Obesity	16 (14.95)	13 (6.37)	29 (9.32)	
**Screen time, %**				<0.001
<2 h/day	57 (53.27)	181 (87.44)	238 (75.80)	
≥2 h/day	50 (46.73)	26 (12.56)	76 (24.20)	
**Physical activity, %**				0.003
High	38 (35.51)	109 (52.66)	147 (46.82)	
Low	23 (21.50)	21 (10.14)	44 (14.01)	
Moderate	46 (42.99)	77 (37.20)	123 (39.17)	

a *1 TD children with missing data on the paternal education.*

b *16 children with ASD failed to complete the Wechsler IV intelligence test due to their low cognitive ability.*

c *3 TD children with missing data on the BMI (body mass index).*

*ASD, Autism Spectrum Disorder; TD, Typical Developing; FSIQ, Full Scale Intelligence Quotient; BMI, Body Mass Index*.

### SSBs, milk intake and emotional and behavioral problems (Strength and Difficulties Questionnaire Scores)

As shown in Table 2 (Supplementary Figure S2), approximately half of the children in both the ASD and TD children consumed at least one time of SSBs each week (66.35 and 59.9%, respectively), there was no significant difference between the two groups in SSBs intake. However, compared to TD children, children with ASD consumed less times of milk (*p* < 0.05). In children with ASD, the scores of the other subscales of SDQ except for prosocial behavior were higher than TD children (*p* < 0.05). The distribution of SSBs, milk intake, and SDQ sub-scales scores in children with ASD and TD children were shown in Supplementary Figures S2, S3.

**Table 2:** The comparison of SSBs and milk intake and SDQ scores between ASD and TD.

	**ASD (*N =* 107)**	**TD (*N =* 207)**	**Total (*N =* 314)**	***p-*value**
**SSBs intake, %**				0.274
0 time/week	36 (33.64)	83 (40.10)	119 (37.90)	
1–2 times/week	51 (47.66)	98 (47.34)	149 (47.45)	
>2 times/week	20 (18.69)	26 (12.56)	46 (14.65)	
**Milk intake, %**				0.033
0–3 time/week	32 (29.91)	37 (17.87)	69 (21.97)	
4–7 times/week	54 (50.47)	112 (54.11)	166 (52.87)	
>7 times/week	21 (19.63)	58 (28.02)	79 (25.16)	
**Emotional symptom** ^a^	2.78 (1.80)	2.39 (2.01)	2.52 (1.95)	0.007
**Conduct problem** ^a^	2.59 (1.99)	2.06 (1.41)	2.24 (1.65)	0.037
**Hyperactivity/Inattention** ^a^	6.64 (2.29)	4.49 (2.59)	5.22 (2.69)	<0.001
**Peer relationship problem** ^a^	5.21 (1.91)	1.82 (1.45)	2.98 (2.28)	<0.001
**Prosocial behavior** ^a^	4.57 (2.26)	6.68 (2.11)	5.96 (2.38)	<0.001

a *The comparison of SDQ scores was adjusted for the following covariates: sex, age, maternal education, paternal education, monthly family income.*

*SSBs, Sugar-sweetened Beverages; ASD, Autism Spectrum Disorder; TD, Typical Developing; SDQ, Strength and Difficulty Questionnaire*.

### Association of SSBs, milk intake, and their interactive effects on emotional and behavioral problems among all participants

As shown in Table 3, higher frequent milk intake was positively associated with prosocial behavior (*p* for trend <0.05) among all participants. The interactive effects of SSBs intake and group in emotional symptom (β = −1.58, 95%CI: −2.93, −0.23, *p* = 0.022) and conduct problem (β = −1.21, 95% CI: −2.35, −0.07, *p* = 0.038) among all participants was significant, and the interactive effects of milk and group in prosocial behavior (β = −1.69, 95% CI: −3.21, −0.17, *p* = 0.029) among all participants was significant. Thus, we further analyzed the association of SSBs, and milk intake with emotional and behavioral problems in the group of children with ASD and TD children respectively. Besides, the crude model showed the same results as the adjust model (See Supplementary Table S1).

**Table 3:** Adjusted model of association of SSBs, milk intake and their interactive effects on emotional and behavioral problems among all participants.

**Adjust model**	**Emotional** **symptom**	**Conduct** **problem**	**Hyperactivity/** **Inattention**	**Peer relationship** **problem**	**Prosocial** **behavior**
	**β (95%*CI*)**	**β (95%*CI*)**	**β (95%*CI*)**	**β (95%*CI*)**	**β (95%*CI*)**
**SSBs**					
**SSBs intake (*****N** **=*** **314)** ^**a**^					
1–2 times/week	−0.14 (−0.97, 0.69)	0.69 (−0.01, 1.38)	0.11 (−0.96, 1.18)	−0.25 (−0.96, 0.45)	0.56 (−0.38, 1.49)
>2 times/week	1.04 (−0.04, 2.12)	0.89 (−0.02, 1.80)	0.02 (−1.38, 1.42)	0.10 (−0.82, 1.03)	−0.35 (−1.57, 0.86)
*p* for trend	0.857	0.830	0.727	0.436	0.960
**Group (*****N** **=*** **314)**^**b**^	−0.42 (−1.26, 0.43)	−0.27 (−0.99, 0.44)	−1.90 (−2.99, −0.80)	−3.33 (−4.05, −2.60)	2.27 (1.32, 3.22)
*p* value	0.332	0.450	0.001	**<0.001**	**<0.001**
SSBs*Group (***N** **=*** **314)**^**c**^					
1–2 times/week*Group times/ week*Group	−0.15 (−1.15, 0.85)	−0.39 (−1.23, 0.45)	0.33 (−0.96, 1.63)	0.36 (−0.50, 1.22)	−0.92 (−2.05, 0.21)
*p-*value	0.769	0.365	0.616	0.410	0.110
>2 times/week*Group	−1.58 (−2.93, −0.23)	−1.21 (−2.35, −0.07)	0.49 (−1.26, 2.24)	−0.24 (−1.39, 0.92)	0.39 (−1.14, 1.91)
*p-*value	**0.022**	**0.038**	0.583	0.688	0.621
**Milk**					
**Milk intake (*****N** **=*** **314)**^**d**^					
4–7 times/week	−0.16 (−1.01, 0.70)	0.70 (−0.02, 1.43)	−0.10 (−1.21, 1.00)	−0.02 (−0.75, 0.71)	0.20 (−0.75, 1.15)
>7 times/week	−1.02 (−2.10, 0.06)	0.31 (−0.62, 1.23)	0.06 (−1.34, 1.47)	−0.70 (−1.62, 0.23)	1.68 (0.47, 2.89)
*p* for trend	0.514	0.954	0.513	0.358	**0.022**
**Group (*****N** **=*** **314)**^**b**^	−0.71 (−1.75, 0.34)	−0.12 (−1.01, 0.77)	−1.03 (−2.39, 0.32)	−3.30 (−4.19, −2.41)	2.73 (1.57, 3.90)
*p-*value	0.185	0.794	0.136	**<0.001**	**<0.001**
Milk*Group (***N** **=*** **314)**^**e**^					
4–7 times/week*Group times/ week*Group	−0.46 (−1.59, 0.66)	−0.81 (−1.76, 0.15)	−0.65 (−2.10, 0.81)	−0.10 (−1.06, 0.86)	−0.77 (−2.03, 0.48)
*p*-value	0.418	0.099	0.385	0.844	0.227
>7 times/week*Group	1.00 (−0.36, 2.37)	−0.48 (−1.64, 0.68)	−0.80 (−2.57, 0.96)	0.65 (−0.52, 1.81)	−1.69 (−3.21, −0.17)
*p-*value	0.149	0.418	0.373	0.275	**0.029**
SSBs*Milk					
SSBs*Milk (***N** **=*** **314)**	−0.01 (−0.08, 0.06)	0.01 (−0.05, 0.07)	0.03 (−0.06, 0.13)	−0.01 (−0.07, 0.05)	0.01 (−0.06, 0.09)
*p-*value	0.762	0.780	0.459	0.803	0.716
**Group (*****N** **=*** **314)**^**b**^	−0.35 (−1.48, 0.77)	−0.14 (−1.10, 0.82)	−1.31 (−2.76, 0.14)	−3.11 (−4.06, −2.15)	2.19 (0.93, 3.44)
*p-*value	0.538	0.776	0.076	**<0.001**	**0.001**
SSBs*Milk*Group (***N** **=*** **314)**^**f**^	0.09 (−0.00, 0.18)	0.03 (−0.05, 0.11)	0.02 (−0.10, 0.14)	0.07 (−0.01, 0.14)	−0.07 (−0.18, 0.03)
*p-*value	0.061	0.447	0.740	0.095	0.164

*β value (95% Confidence Interval) from linear regression represented the correlation between variables and emotional and behavioral problems. Adjusted model was adjusted for sex, age, physical activity, FSIQ, screen time, BMI, maternal education, paternal education, monthly family income.*

*^a^0 time/week of SSBs intake is the reference group.*

*^b^ASD group is the reference group.*

^c^0 time/week^*^Group is the reference group.

*^d^0–3 times/week of milk intake is the reference group.*

^e^0–3 times/week^*^Group is the reference group.

^f^SSBs^*^milk^*^ Group is the reference group.

*SSBs, Sugar-sweetened Beverages; FSIQ, Full Scale Intelligence Quotient; BMI, Body Mass Index; ASD, Autism Spectrum Disorder.*

*The bold values indicate statistically significant association between variables and emotional and behavioral problems*.

### Association of SSBs, milk intake and their interactive effects on emotional and behavioral problems in children with ASD

The relationship between SSBs, milk intake, and their interactive effects on emotional and behavioral problems in children with ASD was presented in Table 4. More frequent of SSBs intake was significantly associated with emotional symptoms in children with ASD, and there were significant dose-response relationships between SSBs intake and emotional symptoms (all *p* for trend <0.05). The crude model estimates (95% *CI*) for emotional symptom across the SSBs categories were −0.18 (−0.94, 0.57) for 1–2 times of SSBs per week, 0.91 (−0.06, 1.88) for >2 times per week. After adjusting covariates, the dose-response relationships between SSBs intake and emotional symptoms still remained, the estimates (95% *CI*) for emotional symptoms across the SSBs categories were −0.08 (−0.85, 0.70) for 1–2 times of SSBs per week, 0.98 (−0.05, 2.00) for >2 times per week.

**Table 4:** Association of SSBs, milk intake and their interactive effects on emotional and behavioral problems in children with ASD.

	**Emotional** **symptom**	**Conduct** **problem**	**Hyperactivity/** **Inattention**	**Peer relationship** **problem**	**Prosocial** **behavior**
	**β (95%*CI*)**	**β (95%*CI*)**	**β (95%*CI*)**	**β (95%*CI*)**	**β (95%*CI*)**
**SSBs intake**
**Crude model (*****N** **=*** **107)**^**a**^
1–2 times/week	−0.18 (−0.94, 0.57)	0.72 (−0.12, 1.56)	0.10 (−0.88, 1.08)	−0.21 (−1.03, 0.61)	0.58 (−0.38, 1.54)
>2 times/week	0.91 (−0.06, 1.88)	0.87 (−0.21, 1.94)	0.32 (−0.94, 1.58)	0.35 (−0.70, 1.40)	−0.36 (−1.59, 0.87)
*p* for trend	**0.045**	0.260	0.614	0.692	0.824
**Adjusted model (*****N** **=*** **107)**^**a**^
1–2 times/week	−0.08 (−0.85, 0.70)	0.58 (−0.29, 1.45)	0.08 (−0.99, 1.15)	−0.15 (−1.04, 0.73)	0.47 (−0.56, 1.50)
>2 times/week	0.98 (−0.05, 2.00)	0.92 (−0.23, 2.08)	0.05 (−1.36, 1.47)	0.06 (−1.11, 1.23)	−0.66 (−2.02, 0.70)
*p* for trend	**0.048**	0.146	0.957	0.876	0.259
**Milk intake**
**Crude model (*****N** **=*** **107)**^**b**^
4–7 times/week	−0.31 (−0.94, 0.57)	0.78 (−0.09, 1.65)	0.01 (−1.00, 1.03)	−0.09 (−0.93, 0.75)	0.18 (−0.78, 1.15)
>7 times/week	−0.99 (−1.98, 0.00)	0.30 (−0.79, 1.39)	−0.25 (−1.53, 1.03)	−0.80 (−1.85, 0.25)	1.60 (0.38, 2.82)
*p* for trend	0.054	0.687	0.704	0.126	**0.009**
**Adjusted model (*****N** **=*** **107)**^**b**^
4–7 times/week	−0.22 (−1.14, 0.70)	0.21 (−0.99, 1.41)	0.56 (−0.49, 1.60)	0.16 (−0.83, 1.16)	0.38 (−0.73, 1.50)
>7 times/week	−0.89 (−1.31, 1.55)	0.12 (−1.99, 0.21)	0.12 (0.43, 3.10)	−0.60 (−1.79, 0.59)	1.76 (−1.13, 1.37)
*p* for trend	0.071	0.616	0.969	0.213	**0.006**
**Interaction**
**Crude model (*****N** **=*** **107)**
SSBs*Milk	−0.00(−0.03,0.03)	0.02(−0.02,0.05)	−0.00(−0.04,0.04)	−0.01(−0.04,0.03)	0.01 (−0.04,0.05)
*p* value	0.936	0.372	0.967	0.698	0.775
**Adjusted model (*****N** **=*** **107)**
SSBs*Milk	−0.00 (−0.04,0.03)	0.02 (−0.02,0.05)	−0.01(−0.06,0.03)	−0.02 (−0.06,0.02)	0.01 (−0.04,0.05)
*p-*value	0.834	0.433	0.638	0.368	0.721

*β value (95% Confidence Interval) from linear regression represented the correlation between variables and emotional and behavioral problems. Adjusted model was adjusted for sex, age, physical activity, FSIQ, screen time, BMI, maternal education, paternal education, monthly family income.*

*^a^0 time/week of SSBs intake was the reference group.*

*^b^0–3 times/week of milk intake was the reference group.*

*SSBs, Sugar-sweetened Beverages; ASD, Autism Spectrum Disorder; FSIQ, Full Scale Intelligence Quotient; BMI, Body Mass Index.*

*The bold values indicate statistically significant association between variables and emotional and behavioral problems*.

Less frequent milk intake was positively associated with prosocial behavior. In children with ASD, there were significant dose-response relationships between milk intake and prosocial behavior (all *p* for trend <0.05). The crude model estimates (95%*CI*) for prosocial behavior across the milk categories were 0.18 (-0.78, 1.15) for 4–7 times of milk intake per week, 1.60 (0.38, 2.82) for >7 times per week. After adjusting covariates, the dose-response relationships between milk intake and prosocial behavior still remained, the estimates (95% *CI*) for prosocial behavior across the milk categories were 0.38 (−0.73, 1.50) for 4–7 times of milk intake per week, 1.76 (−1.13, 1.37) for >7 times per week.

We further assessed the interactive effects of SSBs and milk intake on emotional and behavioral problems in children with ASD. However, no significant interactive effects of SSBs and milk intake on emotional and behavioral problems were observed.

### Association of SSBs, milk intake, and their interactive effects on emotional and behavioral problems in TD children

Table 5 provided detailed information about the association of SSBs, milk intake, and their interactive effects on emotional and behavioral problems in TD children. The results showed no significant differences both in SSBs and milk intake with emotional and behavioral problems. At the same time, there were no interaction effects of SSBs and milk intake on emotional and behavioral problems in TD children.

**Table 5:** Association of SSBs, milk intake and their interactive effects on emotional and behavioral problems in TD children.

	**Emotional** **symptom**	**Conduct** **problem**	**Hyperactivity/** **Inattention**	**Peer relationship** **problem**	**Prosocial** **behavior**
	**β (95%*CI*)**	**β (95%*CI*)**	**β (95%*CI*)**	**β (95%*CI*)**	**β (95%*CI*)**
**SSBs intake**
**Crude model (*****N** **=*** **207)**^**a**^
1–2 times/week	−0.29 (−0.88, 0.29)	0.32 (−0.09, 0.73)	0.57 (−0.19, 1.33)	0.12 (−0.31, 0.55)	0.30 (−0.92, 0.32)
>2 times/week	−0.47 (−1.36, 0.41)	−0.34 (−0.95, 0.28)	0.62 (−0.51, 1.76)	0.14 (−0.50, 0.78)	0.08 (−0.86, 1.01)
*p* for trend	0.288	0.308	0.616	0.669	0.877
**Adjusted model (*****N** **=*** **203)**^**a**^
1–2 times/week	−0.32 (−0.92, 0.29)	0.30 (−0.12, 0.72)	0.43 (−0.33, 1.18)	0.05 (−0.39, 0.49)	−0.31 (−0.96, 0.33)
>2 times/week	−0.60 (−1.55, 0.35)	−0.42 (−1.09, 0.23)	0.33 (−0.86, 1.52)	−0.20 (−0.89, 0.49)	0.11 (−0.90, 1.13)
*p* for trend	0.218	0.217	0.580	0.569	0.835
**Milk intake**
**Crude model (*****N** **=*** **207)**^**b**^
4–7 times/week	−0.83 (−1.58, −0.08)	0.78 (−0.09, 1.65)	−0.07 (−0.61, 0.47)	−0.10 (−0.65, 0.46)	−0.74 (−1.54,0.06)
>7 times/week	−0.34 (−1.17, 0.50)	0.30 (−0.79, 1.39)	−0.05 (−0.64, 0.55)	−0.04 (−0.65, 0.57)	−0.21 (−1.09,0.67)
*p* for trend	0.927	0.953	0.450	0.993	0.738
**Adjusted model (*****N** **=*** **203)**^**b**^
4–7 times/week	−0.70 (−1.48, 0.08)	−0.13 (−0.69, 0.43)	−0.78 (−1.76, 0.21)	−0.12 (−0.69, 0.45)	−0.64 (−1.47,0.20)
>7 times/week	−0.04 (−0.91, 0.84)	−0.19 (−0.82, 0.43)	−0.78 (−1.89, 0.32)	−0.02 (−0.66, 0.62)	0.00 (−0.94, 0.93)
*p* for trend	0.435	0.583	0.320	0.909	0.454
**Interaction**
**Crude model (*****N** **=*** **107)**
SSBs*Milk	0.00 (−0.03, 0.02)	0.00 (−0.01, 0.02)	0.02 (−0.01, 0.04)	0.01 (−0.00, 0.03)	−0.01 (−0.03, 0.01)
*p-*value	0.730	0.964	0.264	0.162	0.321
**Adjusted model (*****N** **=*** **107)**
SSBs*Milk	0.00 (−0.03, 0.02)	0.00 (−0.02, 0.01)	0.01 (−0.02, 0.04)	0.00 (−0.01, 0.02)	−0.01 (−0.04, 0.01)
*p-*value	0.717	0.798	0.604	0.623	0.349

*β value (95% Confidence Interval) from general linear models represented the correlation between variables and emotional and behavioral problems. Adjusted model was adjusted for sex, age, physical activity, FSIQ, screen time, BMI, maternal education, paternal education, monthly family income.*

*SSBs, Sugar-sweetened Beverages; TD, Typical Developing; FSIQ, Full Scale Intelligence Quotient; BMI, Body Mass Index.*

*^a^0 time/week of SSBs intake was the reference group.*

*^b^0–3 times/week of milk intake was the reference group*.

## Discussion

We found that compared to TD children, children with ASD had a higher frequency of milk intake, more serious emotional symptoms, conduct problems, hyperactivity/inattention, peer relationship problems, and less prosocial behavior. Among all participants, the interactive effects of SSBs intake with the group, and milk intake with the group were significant. Thus, we further analyzed the association of SSBs, and milk intake with emotional and behavioral problems in the two groups respectively. In children with ASD, higher frequent SSBs intake was associated with more serious emotional symptoms, and higher frequent milk intake was associated with better prosocial behavior, while there were no significant interactive effects of SSBs and milk intake on emotional and behavioral problems in children with ASD and TD. It was the first study to explore the relationships between SSBs and milk intake with emotional and behavioral problems in children with ASD, which could provide an important basis for clinical nutritional intervention for children with ASD.

In the current study, there was no significant difference in the frequency of SSBs intake between children with ASD and TD children. Inconsistent with our study, some studies showed that compared to TD children, children with ASD tended to intake more SSBs and sugars (10, 11, 38). A study with a large sample among Chinese children aged 6–17 years showed that only 24.50% of the children consumed SSBs <1 time /week, and 25.90% consumed SSBs ≥5 times/week (39). However, in the current study, approximately 40.10% of children with TD never consumed SSBs in the past week, and 12.56% consumed >2 times/week. Most of the population in the current study were from the urban area of Guangzhou in China. Studies showed that due to the warm and humid climate and cultural differences, most people in Guangzhou tend to consume less strong flavored food, which may result in a lower frequency of SSBs intake in the current study population (40). Furthermore, compared to other studies conducted in China, the parental education level in the current population was relatively higher (5). Studies showed that a higher parental education level was associated with a lower frequency of SSBs intake (41).

Interestingly, we found that a higher frequency of SSBs intake was positively associated with emotional problems in children with ASD. A growing body of persuasive evidence posed a positive correlation between SSBs intake and emotional and behavioral problems (5), common mental disorders, and depression (6, 42). Several potential biological mechanisms might explain the association. Because SSBs were the principal source of added sugar in diets (43), it was reasonable to propose that the mechanism for explaining the association between SSBs intake and the emotional symptom was relevant to sugar content. First, several studies indicated that sugar intake resulted in a high risk of inflammation (44, 45). A cohort study indicated that long-term inflammation increased the risk of common mental disorders (46), such as depression. What's more, studies showed that inflammation was positively associated with psychological problem and autism symptoms (47). Thus, sugar intake triggered inflammation in children with ASD, which might be responsible for the association between SSBs intake and emotional symptom. Additionally, additives in SSBs, such as artificial food colors (AFCs) and caffeine, might somewhat explain the association between SSBs intake and emotional problem. Researches showed that AFCs had deleterious effects on children' s behavior (48) and caffeine intake was positively correlated with the severity of depression and insomnia in adolescents (49). In TD children, we found no association between SSBs intake with emotional and behavioral problems, however, there was no significant difference in SSBs intake between children with ASD and TD children. The results showed that compared to TD children, children with ASD were more vulnerable to the SSBs. Literature indicated that as one of the major environmental factors influencing children with ASD, diet may influence gastrointestinal microbiota composition and nutrients deficiency, then lead to the disorder of intestinal brain axis (50).

Unfortunately, consistent with previous studies (24, 25, 51, 52), results of our study indicated that children with ASD displayed lower milk intake than TD children. It's unclear why children with ASD had lower milk intake than TD children. Potential explanations might be included: first, compared to TD children, the children with ASD had lower socioeconomic status (parental education, monthly family income) in this study. Studies showed that higher family income and parental education levels have a positive relationship with milk intake (53, 54). Second, the literature showed that approximately 90% of children with ASD experienced a string of feeding-related problems (55), and they were more likely to experience food allergies including milk/dairy, nuts, and fruits (56). Therefore, the parents of children with ASD might take the initiative to reduce their milk intake for autistic children.

The current study showed that higher frequent milk intake was positively associated with better prosocial behavior in children with ASD but not in TD children. The positive association between milk intake and prosocial behavior might be explained by several mechanisms. First, milk is an important source of macronutrients and micronutrients in the diets of children and adolescents, including protein, vitamins, and minerals (57), and plays important role in meeting multiple nutrient recommendations (58). Compared to TD children, children with ASD had less frequent milk intake, which might further result in a lack of macronutrients and micronutrients in these children. Children with ASD have impaired development and are more vulnerable to nutrient deficiencies (59). A meta-analysis showed that vitamin/mineral supplementation can reduce anti-social behavior (60). Secondly, calcium and dairy products in milk may decrease inflammation in children with ASD. Evidence from mice and human demonstrated that dietary calcium decreased oxidative and inflammatory stress *in vivo* and dairy products contain additional factors, such as angiotensin-converting enzyme inhibitors, which might further suppress oxidative and inflammatory stress (61). In addition, studies indicated that higher frequent milk intake was associated with better perceived happiness, and sleep satisfaction (62), which may help children increase prosocial behavior. We found that higher frequent milk intake showed relatively lower risk of prosocial problem in children with ASD, while there were no significant correlation between milk intake and prosocial problem in TD children. Future studies may be needed to further explore the benefits of milk for children and the effects of dietary patterns on emotional and behavioral problems in addition to milk.

Several studies showed the association between SSBs and milk intake, reporting that the increased frequency of SSBs intake was coupled with the decreased intake of milk among school-aged children (14, 63). The current study showed that there were no interactive effects of SSBs and milk intake on emotional and behavioral problems in children with ASD and TD, which suggested that SSBs and milk intake have independent effects on emotional and behavioral problems. It was the first study conducted on the interactive effects of SSBs and milk intake on emotional and behavioral problems in children with ASD and TD, more researches need to further explore the interactive effects of SSBs and milk intake on emotional and behavioral problems in the future.

However, this study had several limitations. First, the current study was cross-sectional and cannot verify a causal relationship between SSBs and milk intake with emotional and behavioral problems. Future studies should investigate the longitudinal effects of SSBs and milk intake on emotional and behavioral problems in children with ASD. Secondly, the data concerning the SSBs and milk intake, SDQ, and questionnaire-based sociodemographic factors were reported by parents and guardians, which might be influenced by recall bias. Furthermore, there were many factors influencing SDQ and we only have explored a part of them, so further researches need to pay attention to other factors.

In summary, this study suggested that compared to TD children, children with ASD have less frequent milk intake and worse performance on all SDQ subscales. Furthermore, in children with ASD but not in TD children, higher frequency of SSBs intake was positively associated with an emotional problem, higher frequent milk intake was associated with better prosocial behavior, and there were no interactive effects of SSBs and milk intake on emotional and behavioral problems both in children with ASD and TD. We speculated that compared to TD children, the emotional problem and prosocial behavior of children with ASD were more vulnerable due to the SSBs and milk intake. However, the current study was a cross-sectional design, and prospective research is needed to verify our hypothesis in the future.

## Data availability statement

The raw data supporting the conclusions of this article will be made available by the authors, without undue reservation.

## Ethics statement

The studies involving human participants were reviewed and approved by Sun Yat-sen University. Written informed consent to participate in this study was provided by the participants' legal guardian/next of kin.

## Author contributions

LL designed the study. NP, XX, HL, JC, CJ, and SP performed the survey research. NP and ST analyzed the data. ST drafted the manuscript. XL and JJ were critical revision of the manuscript for important intellectual content. All authors contributed to the article and approved the submitted version.

## Funding

This work was supported by the Key-Area Research and Development Program of Guangdong Province (2019B030335001) and the National Natural Science Foundation of China (81872639).

## Conflict of interest

The authors declare that the research was conducted in the absence of any commercial or financial relationships that could be construed as a potential conflict of interest.

## Publisher's note

All claims expressed in this article are solely those of the authors and do not necessarily represent those of their affiliated organizations, or those of the publisher, the editors and the reviewers. Any product that may be evaluated in this article, or claim that may be made by its manufacturer, is not guaranteed or endorsed by the publisher.

## References

[1] Kodak T Bergmann S. Autism spectrum disorder: characteristics, associated behaviors, and early intervention. Pediatr Clin North Am. (2020) 67:525–35. 10.1016/j.pcl.2020.02.007 32443991

[2] Yorke I White P Weston A Rafla M Charman T Simonoff E. The association between emotional and behavioral problems in children with autism spectrum disorder and psychological distress in their parents: a systematic review and meta-analysis. J Autism Dev Disord. (2018) 48:3393–415. 10.1007/s10803-018-3605-y 29777471 PMC6153902

[3] Ooi YP Tan ZJ Lim CX Goh TJ Sung M. Prevalence of behavioural and emotional problems in children with high-functioning autism spectrum disorders. Aust N Z J Psychiatry. (2011) 45:370–5. 10.3109/00048674.2010.534071 21138332

[4] Hill AP Zuckerman KE Hagen AD Kriz DJ Duvall SW van Santen J. Aggressive behavior problems in children with autism spectrum disorders: prevalence and correlates in a large clinical sample. Res Autism Spectr Disord. (2014) 8:1121–33. 10.1016/j.rasd.2014.05.006 25221619 PMC4160737

[5] Geng M Jiang L Wu X Ding P Liu W Liu M. Sugar-sweetened beverages consumption are associated with behavioral problems among preschoolers: a population based cross-sectional study in China. J Affect Disord. (2020) 265:519–25. 10.1016/j.jad.2020.01.076 32090780

[6] Knuppel A Shipley MJ Llewellyn CH Brunner EJ. Sugar intake from sweet food and beverages, common mental disorder and depression: prospective findings from the Whitehall II study. Sci Rep. (2017) 7:6287. 10.1038/s41598-017-05649-7 28751637 PMC5532289

[7] Schwartz DL Gilstad-Hayden K Carroll-Scott A Grilo SA McCaslin C Schwartz M. Energy drinks and youth self-reported hyperactivity/inattention symptoms. Acad Pediatr. (2015) 15:297–304. 10.1016/j.acap.2014.11.006 25676784 PMC4772143

[8] Zhuang X Liu Y Gittelsohn J Lewis E Song S Ma Y. Sugar-sweetened beverages consumption and associated factors among Northeastern Chinese children. Nutrients. (2021) 13:2233. 10.3390/nu13072233 34209665 PMC8308402

[9] Scharf RJ DeBoer MD. Sugar-sweetened beverages and children's health. Annu Rev Public Health. (2016) 37:273–93. 10.1146/annurev-publhealth-032315-021528 26989829

[10] Canals-Sans J Esteban-Figuerola P Morales-Hidalgo P Arija V. Do Children with autism spectrum disorders eat differently and less adequately than those with subclinical ASD and typical development? EPINED Epidemiological Study. J Autism Dev Disord. (2021) 52:361–75. 10.1007/s10803-021-04928-7 33745113

[11] Raspini B Prosperi M Guiducci L Santocchi E Tancredi R Calderoni S. Dietary patterns and weight status in Italian preschoolers with autism spectrum disorder and typically developing children. Nutrients. (2021) 13:4039. 10.3390/nu13114039 34836294 PMC8617730

[12] Currais A Farrokhi C Dargusch R Goujon-Svrzic M Maher P. Dietary glycemic index modulates the behavioral and biochemical abnormalities associated with autism spectrum disorder. Mol Psychiatry. (2016) 21:426–36. 10.1038/mp.2015.64 26055422

[13] Mathias KC Slining MM Popkin BM. Foods and beverages associated with higher intake of sugar-sweetened beverages. Am J Prev Med. (2013) 44:351–7. 10.1016/j.amepre.2012.11.036 23498100 PMC3601585

[14] Keller KL Kirzner J Pietrobelli A St-Onge MP Faith MS. Increased sweetened beverage intake is associated with reduced milk and calcium intake in 3- to 7-year-old children at multi-item laboratory lunches. J Am Diet Assoc. (2009) 109:497–501. 10.1016/j.jada.2008.11.030 19248869 PMC2748414

[15] Mrdjenovic G Levitsky DA. Nutritional and energetic consequences of sweetened drink consumption in 6- to 13-year-old children. J Pediatr. (2003) 142:604–10. 10.1067/mpd.2003.200 12838186

[16] Campmans-Kuijpers MJ Singh-Povel C Steijns J Beulens JW. The association of dairy intake of children and adolescents with different food and nutrient intakes in the Netherlands. BMC Pediatr. (2016) 16:2. 10.1186/s12887-015-0524-3 26749195 PMC4707007

[17] Charvet A Huffman FG. Beverage intake and its effect on body weight status among WIC preschool-age children. J Obes. (2019) 2019:3032457. 10.1155/2019/3032457 30800480 PMC6360070

[18] Dor C Stark AH Dichtiar R Keinan-Boker L Shimony T Sinai T. Milk and dairy consumption is positively associated with height in adolescents: results from the Israeli National Youth Health and Nutrition Survey. Eu J Nutr. (2022). 61:429–38. 10.1007/s00394-021-02661-6 34406484

[19] Louie JCY Flood VM Hector DJ Rangan AM Gill TP. Dairy consumption and overweight and obesity: a systematic review of prospective cohort studies. Obesity Reviews. (2011) 12:E582–92. 10.1111/j.1467-789X.2011.00881.x 21521450

[20] Jin S Je Y. Dairy Consumption and Risk of Metabolic Syndrome: Results from Korean Population and Meta-Analysis. Nutrients. (2021) 13. 10.3390/nu13051574 34066690 PMC8151357

[21] Rangan AM Flood VL Denyer G Ayer JG Webb KL Marks GB. The effect of dairy consumption on blood pressure in mid-childhood: CAPS cohort study. Eur J Clin Nutr. (2012) 66:652–7. 10.1038/ejcn.2011.218 22234043

[22] Gomez AL Kraemer WJ Maresh CM Lee EC Szivak TK Caldwell LK. Resistance training and milk-substitution enhance body composition and bone health in adolescent girls. J Am Coll Nutr. (2021) 40:193–210. 10.1080/07315724.2020.1770636 32521207

[23] Grandner MA Jackson N Gerstner JR Knutson KL. Sleep symptoms associated with intake of specific dietary nutrients. J Sleep Res. (2014) 23:22–34. 10.1111/jsr.12084 23992533 PMC3866235

[24] Diolordi L del Balzo V Bernabei P Vitiello V Donini LM. Eating habits and dietary patterns in children with autism. Eat Weight Disord-Stud Anorex. (2014) 19:295–301. 10.1007/s40519-014-0137-0 24981567

[25] Herndon AC DiGuiseppi C Johnson SL Leiferman J Reynolds A. Does nutritional intake differ between children with autism spectrum disorders and children with typical development? J Autism Dev Disord. (2009) 39:212–22. 10.1007/s10803-008-0606-2 18600441

[26] Loewen OK Maximova K Ekwaru JP Faught EL Asbridge M Ohinmaa A. Lifestyle behavior and mental health in early adolescence. Pediatrics. (2019) 143:e20183307. 10.1542/peds.2018-3307 31004047

[27] Wu X Veugelers PJ Ohinmaa A. Health behavior, health-related quality of life, and mental health among Canadian children: a population-based cohort study. Front Nutr. (2021) 8:638259. 10.3389/fnut.2021.638259 33777992 PMC7991792

[28] Lv YJ LL Li DL Cai L Zhu YN Feng CY Tan WQ Chen YJ. Reliability and validity of the questionnaire of surveillance on students' constitution and health for primary school students in Guangzhou. Matern Child Health Care Chin. (2020) 35:1511–6. 10.19829/j.zgfybj.issn.1001-4411.2020.08.047

[29] Goodman R Renfrew D Mullick M. Predicting type of psychiatric disorder from Strengths and Difficulties Questionnaire (SDQ) scores in child mental health clinics in London and Dhaka. Eur Child Adolesc Psychiatry. (2000) 9:129–34. 10.1007/s007870050008 10926063

[30] Theunissen MH Vogels AG de Wolff MS Reijneveld SA. Characteristics of the strengths and difficulties questionnaire in preschool children. Pediatrics. (2013) 131:e446–54. 10.1542/peds.2012-0089 23296429

[31] Mazarello Paes V Hesketh K O'Malley C Moore H Summerbell C Griffin S. Determinants of sugar-sweetened beverage consumption in young children: a systematic review. Obes Rev. (2015) 16:903–13. 10.1111/obr.12310 26252417 PMC4737242

[32] Elsabbagh M. Linking risk factors and outcomes in autism spectrum disorder: is there evidence for resilience? BMJ. (2020) 368:l6880. 10.1136/bmj.l6880 31992555

[33] Zeidan J Fombonne E Scorah J Ibrahim A Durkin MS Saxena S. Global prevalence of autism: a systematic review update. Autism Res. (2022) 15:778–90. 10.1002/aur.2696 35238171 PMC9310578

[34] HC Z. Revision of the Chinese version of Wechsler Intelligence Scale for children, Fourth vision. Pychol Sci. (2009) 32:1177–9. 10.16719/j.cnki.1671-6981.2009.05.026

[35] Craig CL Marshall AL Sjöström M Bauman AE Booth ML Ainsworth BE. International physical activity questionnaire: 12-country reliability and validity. Med Sci Sports Exerc. (2003) 35:1381–95. 10.1249/01.MSS.0000078924.61453.FB 12900694

[36] China HISi. Screening for Overweight Obesity Among School-Age Children Adolescents. (2018). Available online at: http://www.nhc.gov.cn/wjw/pqt/201803/a7962d1ac01647b9837110bfd2d69b26.shtml2018 (accessed March 30, 2018).

[37] Mundry R Fischer J. Use of statistical programs for nonparametric tests of small samples often leads to incorrect P values: examples fromAnimal Behaviour. Anim Behav. (1998) 56:256–9. 10.1006/anbe.1998.0756 9710485

[38] Evans EW Must A Anderson SE Curtin C Scampini R Maslin M. Dietary patterns and body mass index in children with autism and typically developing children. Res Autism Spectr Disord. (2012) 6:399–405. 10.1016/j.rasd.2011.06.014 22936951 PMC3427936

[39] Gan Q Xu P Yang T Cao W Xu J Li L. Sugar-sweetened beverage consumption status and its association with childhood obesity among chinese children aged 6-17 years. Nutrients. (2021) 13:2211. 10.3390/nu13072211 34199097 PMC8308281

[40] Zhang T Cai L Ma L Jing J Chen Y Ma J. The prevalence of obesity and influence of early life and behavioral factors on obesity in Chinese children in Guangzhou. BMC Public Health. (2016) 16:954. 10.1186/s12889-016-3599-3 27613102 PMC5016860

[41] Irwin BR Speechley MR Gilliland JA. Assessing the relationship between water and nutrition knowledge and beverage consumption habits in children. Public Health Nutr. (2019) 22:3035–48. 10.1017/S1368980019000715 31084651 PMC10260567

[42] Lien L Lien N Heyerdahl S Thoresen M Bjertness E. Consumption of soft drinks and hyperactivity, mental distress, and conduct problems among adolescents in Oslo, Norway. Am J Public Health. (2006) 96:1815–20. 10.2105/AJPH.2004.059477 17008578 PMC1586153

[43] Huth PJ Fulgoni VL Keast DR Park K Auestad N. Major food sources of calories, added sugars, and saturated fat and their contribution to essential nutrient intakes in the U.S. diet: data from the National Health and Nutrition Examination Survey (2003-2006). Nutr J. (2013) 12:116. 10.1186/1475-2891-12-116 23927718 PMC3751311

[44] Hsu TM Konanur VR Taing L Usui R Kayser BD Goran MI. Effects of sucrose and high fructose corn syrup consumption on spatial memory function and hippocampal neuroinflammation in adolescent rats. Hippocampus. (2015) 25:227–39. 10.1002/hipo.22368 25242636

[45] Spagnuolo MS Iossa S Cigliano L. Sweet but bitter: focus on fructose impact on brain function in rodent models. Nutrients. (2020) 13:1. 10.3390/nu13010001 33374894 PMC7821920

[46] Kivimäki M Shipley MJ Batty GD Hamer M Akbaraly TN Kumari M. Long-term inflammation increases risk of common mental disorder: a cohort study. Mol Psychiatry. (2014) 19:149–50. 10.1038/mp.2013.35 23568195 PMC3903110

[47] Theoharides TC Asadi S Patel AB. Focal brain inflammation and autism. J Neuroinflammation. (2013) 10:46. 10.1186/1742-2094-10-46 23570274 PMC3626551

[48] Arnold LE Lofthouse N Hurt E. Artificial food colors and attention-deficit/hyperactivity symptoms: conclusions to dye for. Neurotherapeutics. (2012) 9:599–609. 10.1007/s13311-012-0133-x 22864801 PMC3441937

[49] Jin MJ Yoon CH Ko HJ Kim HM Kim AS Moon HN. The relationship of caffeine intake with depression, anxiety, stress, and sleep in Korean adolescents. Korean J Fam Med. (2016) 37:111–6. 10.4082/kjfm.2016.37.2.111 27073610 PMC4826990

[50] Berding K Donovan SM. Diet can impact microbiota composition in children with autism spectrum disorder. Front Neurosci. (2018) 12:515. 10.3389/fnins.2018.00515 30108477 PMC6079226

[51] Marí-Bauset S Llopis-González A Zazpe I Marí-Sanchis A Morales Suárez-Varela M. Comparison of nutritional status between children with autism spectrum disorder and typically developing children in the Mediterranean Region (Valencia, Spain). Autism. (2017) 21:310–22. 10.1177/1362361316636976 27132010

[52] Neumeyer AM Cano Sokoloff N McDonnell EI Macklin EA McDougle CJ Holmes TM. Nutrition and bone density in boys with autism spectrum disorder. J Acad Nutr Diet. (2018) 118:865–77. 10.1016/j.jand.2017.11.006 29409733 PMC5924619

[53] Vue H Reicks M. Individual and environmental influences on intake of calcium-rich food and beverages by young Hmong adolescent girls. J Nutr Educ Behav. (2007) 39:264–72. 10.1016/j.jneb.2007.03.092 17826346

[54] Olson BH Chung KR Reckase M Schoemer S. Parental influences on dairy intake in children, and their role in child calcium-fortified food use. J Nutr Educ Behav. (2009) 41:53–7. 10.1016/j.jneb.2008.03.005 19161921

[55] Sharp WG Berry RC McCracken C Nuhu NN Marvel E Saulnier CA. Feeding problems and nutrient intake in children with autism spectrum disorders: a meta-analysis and comprehensive review of the literature. J Autism Dev Disord. (2013) 43:2159–73. 10.1007/s10803-013-1771-5 23371510

[56] Lyall K Van de Water J Ashwood P Hertz-Picciotto I. Asthma and allergies in children with autism spectrum disorders: results from the CHARGE study. Autism Res. (2015) 8:567–74. 10.1002/aur.1471 25722050 PMC6900397

[57] Dror DK Allen LH. Dairy product intake in children and adolescents in developed countries: trends, nutritional contribution, and a review of association with health outcomes. Nutr Rev. (2014) 72:68–81. 10.1111/nure.12078 24330063

[58] Saito A Okada E Tarui I Matsumoto M Takimoto H. The association between milk and dairy products consumption and nutrient intake adequacy among Japanese adults: analysis of the 2016 national health and nutrition survey. Nutrients. (2019) 11:2361. 10.3390/nu11102361 31623382 PMC6835801

[59] Perrin JM Coury DL Hyman SL Cole L Reynolds AM Clemons T. Complementary and alternative medicine use in a large pediatric autism sample. Pediatrics. (2012) 130 Suppl 2:S77–82. 10.1542/peds.2012-0900E 23118257

[60] Benton D. The impact of diet on anti-social, violent and criminal behaviour. Neurosci Biobehav Rev. (2007) 31:752–74. 10.1016/j.neubiorev.2007.02.002 17433442

[61] Zemel MB Sun X. Dietary calcium and dairy products modulate oxidative and inflammatory stress in mice and humans. J Nutr. (2008) 138:1047–52. 10.1093/jn/138.6.1047 18492832

[62] Park S Rim SJ Lee JH. Associations between dietary behaviours and perceived physical and mental health status among Korean adolescents. Nutr Diet. (2018) 75:488–93. 10.1111/1747-0080.12444 29978549

[63] Lasater G Piernas C Popkin BM. Beverage patterns and trends among school-aged children in the US, 1989-2008. Nutr J. (2011) 10:103. 10.1186/1475-2891-10-103 21962086 PMC3196913

