# Supplementary material for: The association between sugar-sweetened beverages and milk intake with emotional and behavioral problems in children with autism spectrum disorder
Source: Front Nutr. 2022 Aug 4;9:927212. doi: 10.3389/fnut.2022.927212 (PMC9386187; doi:10.3389/fnut.2022.927212)
Supplement: Supplementary file 1 [file Table_1.pdf]

Table S1. Crude model of association of SSBs, milk intake and their interactive effects on emotional and behavioral problems among all participants

Crude model	Emotional symptom	Conduct problem	Hyperactivity/Inattention	Peer relationship problem	Prosocial behavior
	β **(95%*CI*)**	***β*** **(95%*CI*)**	***β*** **(95%*CI*)**	***β*** **(95%*CI*)**	***β*** **(95%*CI*)**
**SSBs**					
**SSBs intake (N=314)** [a]					
1-2 times/week	-0.18(-1.01, 0.64)	0.72(0.03, 1.41)	0.10(-0.96, 1.16)	-0.21(-0.90, 0.48)	0.58(-0.34, 1.50)
>2 times/week	0.91(-0.15, 1.96)	0.87(-0.02, 1.75)	0.32(-1.04, 1.69)	0.35(-0.54, 1.24)	-0.36(-1.54, 0.82)
p for trend	0.639	0.538	0.090	0.078	0.289
**Group (N=314)** [b]	-0.10(-0.86, 0.65)	-0.13(-0.76, 0.50)	-2.38(-3.36, -1.41)	-3.50(-4.14, -2.87)	2.45(1.60, 3.29)
p value	0.332	0.450	0.001	**<0.001**	**<0.001**
**SSBs*Group (N=314)** [c]					
1-2 times/week*Group	-0.11(-1.11, 0.89)	-0.40(-1.23, 0.44)	0.47(-0.82, 1.76)	0.33(-0.51, 1.17)	-0.88(-2.00, 0.24)
p value	0.830	0.352	0.475	0.440	0.124
>2 times/week*Group	-1.38(-2.74, -0.02)	-1.20(-2.34, -0.07)	0.30(-1.45, 2.05)	-0.21(-1.35, 0.93)	0.44(-1.08, 1.96)
p value	**0.046**	**0.038**	0.735	0.715	0.571
**Milk**					
**Milk intake (N=314)** [d]					
4-7 times/week	-0.31(-1.16, 0.54)	0.78(0.06, 1.50)	0.01(-1.09, 1.11)	-0.09(-0.81, 0.62)	0.18(-0.76, 1.12)
>7 times/week	-0.99(-2.05, 0.08)	0.30(-0.60, 1.20)	-0.25(-1.63, 1.14)	-0.80(-1.70, 0.10)	1.60(0.42, 2.78)
p for trend	0.232	0.908	0.253	0.131	**0.025**
**Group (N=314)** [b]	-0.19(-1.12, 0.74)	-0.01(-0.80, 0.77)	-1.73(-2.94, -0.53)	-3.53(-4.32, -2.75)	2.98(1.95, 4.01)
p value	0.695	0.971	**0.005**	**<0.001**	**<0.001**
**Milk*Group (N=314)** [e]					
4-7 times/week*Group	-0.52(-1.63, 0.60)	-0.85(-1.80, 0.09)	-0.54(-2.00, 0.91)	-0.00(-0.95, 0.94)	-0.92(-2.16, 0.32)
p value	0.363	0.078	0.463	0.993	0.144
>7 times/week*Group	0.65(-0.69, 1.98)	-0.34(-1.48, 0.79)	-0.31(-2.05, 1.42)	0.76(-0.37, 1.89)	-1.81(-3.30, -0.33)
p value	0.343	0.550	0.723	0.187	**0.017**
**SSBs*Milk**					
**SSBs*Milk (N=314)**	-0.01(-0.08, 0.06)	0.01(-0.05, 0.07)	0.04(-0.05, 0.13)	-0.00(-0.06, 0.05)	0.01(-0.06, 0.09)
p value	0.792	0.711	0.352	0.883	0.737
**Group (N=314)** [b]	-0.01(-1.02, 1.01)	-0.18(-1.04, 0.68)	-2.12(-3.42, -0.82)	-3.45(-4.30, -2.60)	2.34(1.21, 3.46)
p value	0.992	0.676	**0.001**	**<0.001**	**<0.001**
**SSBs*Milk*Group (N=314)** [f]	0.08(-0.01, 0.17)	0.02(-0.06, 0.10)	-0.01(-0.13, 0.11)	0.05(-0.02, 0.13)	-0.07(-0.17, 0.03)
p value	0.086	0.579	0.891	0.172	0.171

β value (95% Confidence Interval) from linear regression represented the correlation between variables and emotional and behavioral problems. [a]: 0 time/week of SSBs intake is the reference group; [b]: ASD group is the reference group; [c]: 0 time/week*Group is the reference group; [d]: 0-3 times/week of milk intake is the reference group; [e]: 0-3 times/week*Group is the reference group; [f]: SSBs*milk*Group is the reference group. SSBs: Sugar-sweetened Beverages; FSIQ: Full Scale Intelligence Quotient; BMI: Body Mass Index; ASD: Autism Spectrum Disorder.
