# Supplementary figures and images for: The association between sugar-sweetened beverages and milk intake with emotional and behavioral problems in children with autism spectrum disorder
Source: Front Nutr. 2022 Aug 4;9:927212. doi: 10.3389/fnut.2022.927212 (PMC9386187; doi:10.3389/fnut.2022.927212)

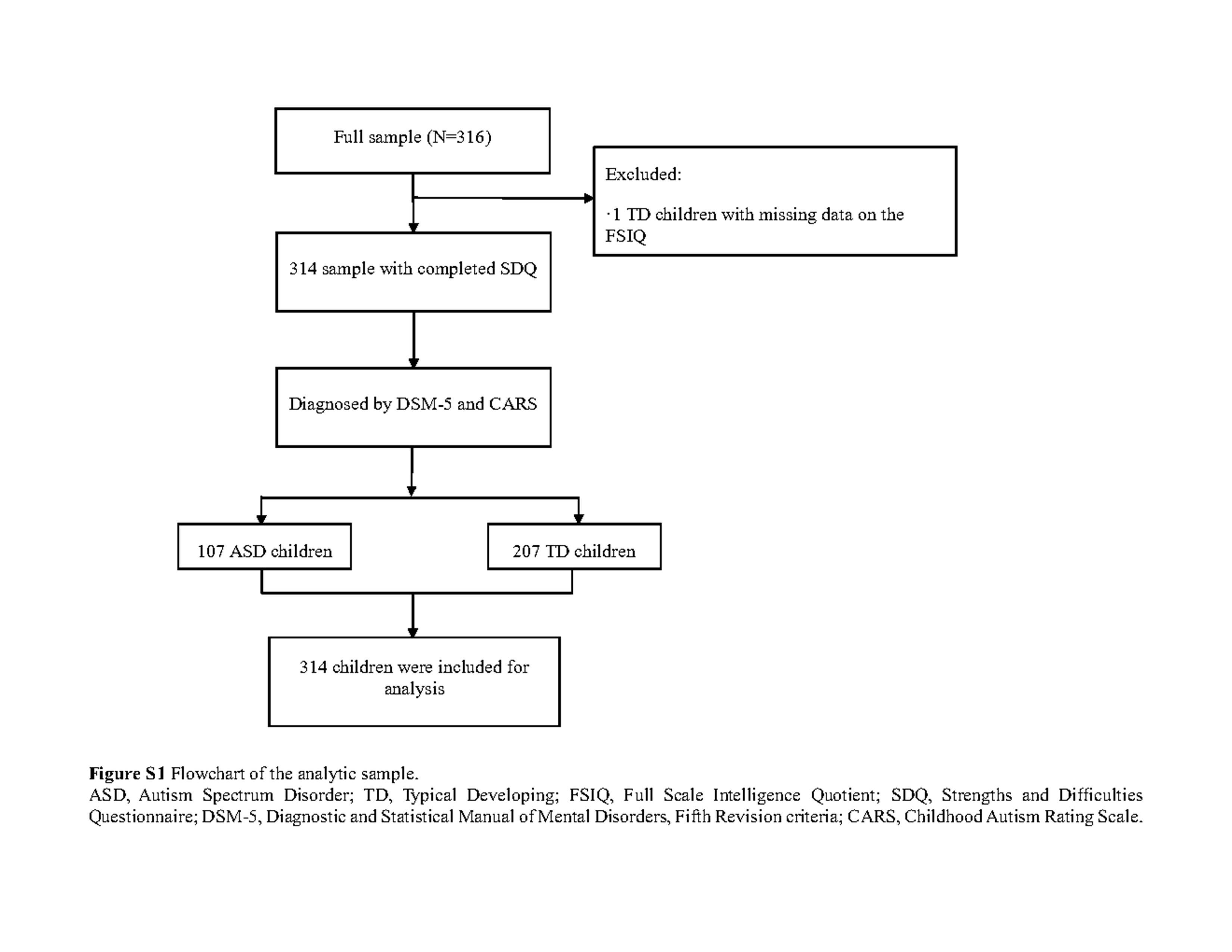

Supplement: Supplementary file 2 [file Image_1.tiff]

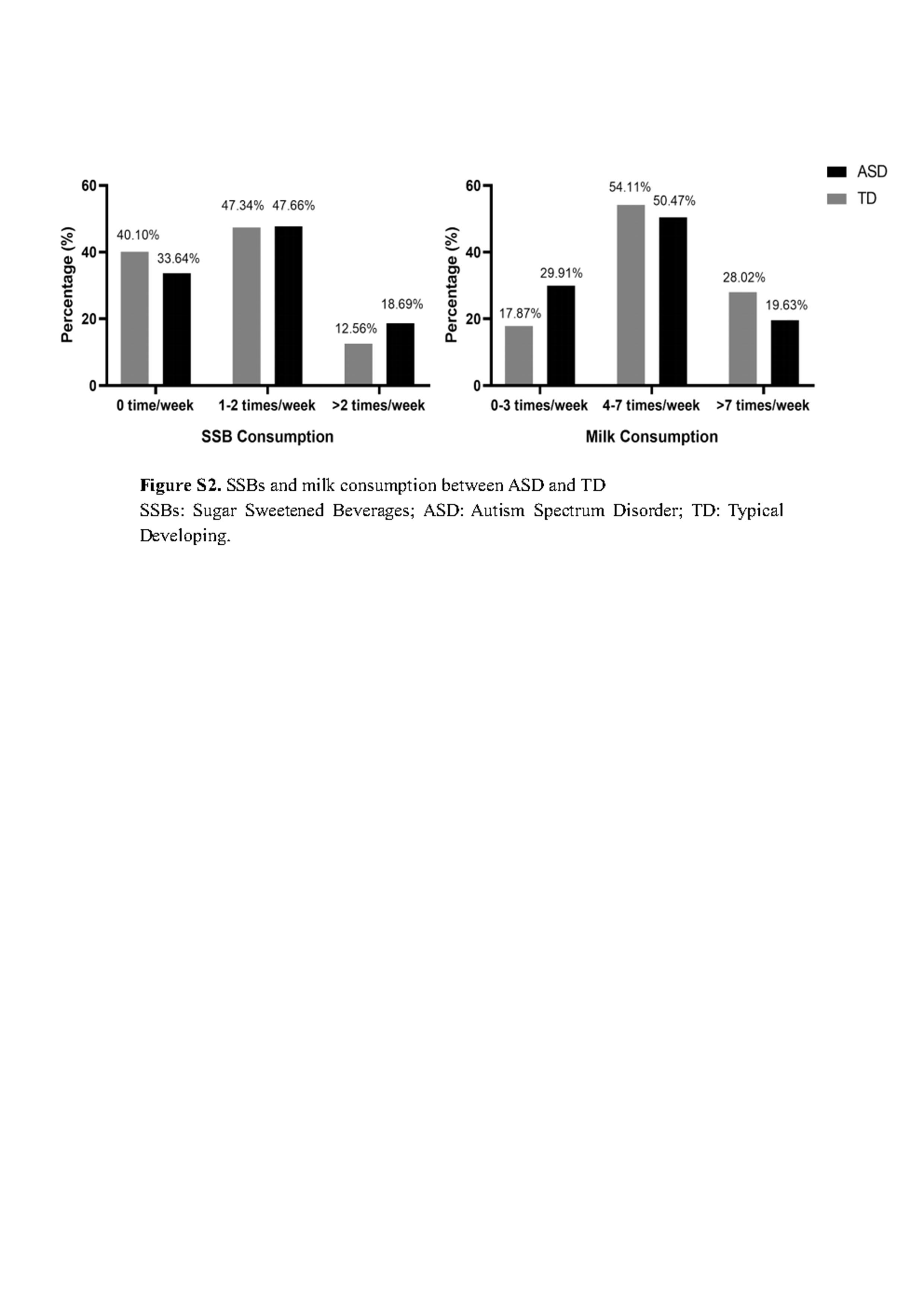

Supplement: Supplementary file 3 [file Image_2.tiff]

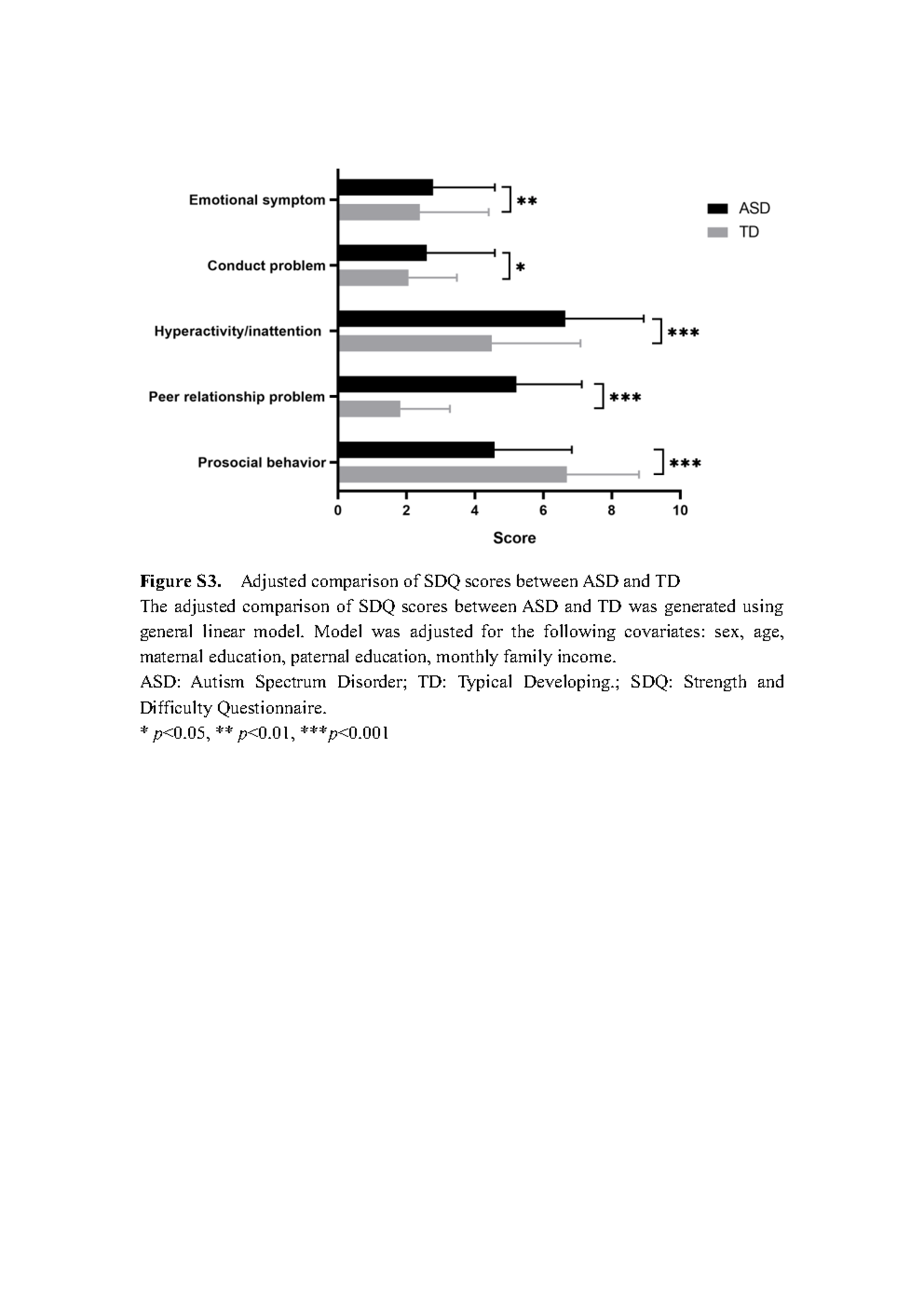

Supplement: Supplementary file 4 [file Image_3.tiff]
